# Association of frailty with physical activity behaviour and well-being in older employees: moderated mediation by functional difficulty

**DOI:** 10.1186/s12889-025-21596-9

**Published:** 2025-01-31

**Authors:** Emelia Danquah, Nestor Asiamah, Reginald Arthur-Mensah Jnr, Alex Sui Wing Chan, Hafiz T. A. Khan

**Affiliations:** 1 Research Directorate, Koforidua Technical University, Koforidua, E/R Ghana; 2 Division of Interdisciplinary Research and Practice, University of Essex, School of Health and Social Care, Colchester, Essex CO4 3SQ UK; 3 Department of Gerontology and Geriatrics, Africa Centre for Epidemiology, P. O. Box AN, 18462 Accra North, Accra, Ghana; 4 Department of Nursing and Midwifery, Faculty of Health and Allied Sciences, Pentecost University, P.O. Box KN 1739, Accra, Ghana; 5 Department of Applied Social Sciences, The Hong Kong Polytechnic University, HJ402, 4/F, Hung Hom, Kowloon, Hong Kong; 6 College of Nursing, Midwifery, and Healthcare, University of West London, Paragon House, Boston Manor Road, Brentford, TW8 9GB UK

**Keywords:** Frailty, Functional difficulty, Older adults, Income, Physical well-being, Physical activity

## Abstract

**Background:**

Many studies have highlighted the association between frailty, physical activity behaviour (PAB), and well-being, but no study has investigated a potential moderated mediating role of functional difficulty in this relationship. This association may not be the same between different income levels. This study, therefore, assessed the above moderated mediation between low- and higher-income samples.

**Methods:**

This research employed a cross-sectional design in accordance with established research-reporting guidelines. The study population comprised two distinct Ghanaian samples, with *N* = 942 individuals in the low-income group and *N* = 600 individuals in the higher income group. Data analysis was carried out using Hayes's Process model through structural equation modelling, with additional sensitivity analyses performed through hierarchical linear regression.

**Results:**

Frailty had a direct negative effect and an indirect negative effect (through functional difficulty) on well-being in both samples. A partial mediation of functional difficulty was found in the relationship between frailty and well-being in both samples. We also found evidence of a moderated mediation by functional difficulty in both samples; however, this effect was stronger in the higher-income sample.

**Conclusion:**

Older employees with frailty are less likely to report lower functional difficulty and well-being at higher PAB. Our results suggest a need for workplace programmes aimed at encouraging PAB. It also reinforces the importance of individuals performing and maintaining PAB.

**Supplementary Information:**

The online version contains supplementary material available at 10.1186/s12889-025-21596-9.

## Introduction

Frailty is a major public health concern because it increases the risk of disability, mobility limitations, dependency, and social isolation. Frailty may discourage health-seeking behaviours such as healthcare utilisation, which is why frail individuals are vulnerable to chronic conditions such as diabetes, hypertension, and kidney disease [1, 2]. There is a consensus among researchers [3, 4] that frailty is an outcome of ageing, so it increases with the individual’s age. As global population ageing intensifies, the global burden of frailty and its associated conditions (e.g., arthritis, osteoporosis, and diabetes) is expected to increase. Healthcare needs driven by frailty are also expected to increase in the older adult population.

Frailty in a clinical context is limitations and impairments in physical performance including Activities of Daily Living (ADLs) and Instrumental Activities of Daily Living (IADLs) [5, 6]. ADLs are daily self-care activities such as toileting, bathing, and eating whereas IADLs are more complex activities (e.g., vising a grocery shop) by which individuals live in their communities [7]. Though several other definitions of frailty exist, we adopt the foregoing definition in this study because it is consistent with our clinical measure of frailty. We aimed to operationalise frailty from a clinical perspective to discuss implications for clinical practice, given that clinical interventions to frailty amidst population ageing may be more reliable and feasible. As this definition suggests, frailty accompanies physical functional difficulty, which we define as the extent to which the individual finds it difficult to perform physical tasks including ADLs and IADLs. Research has shown that functional difficulty is an outcome of frailty [8, 9], suggesting that frail persons would find it more difficult to perform physical tasks.

Deductively, frailty can create feelings of poor health and, therefore, influence poor well-being directly or indirectly by predicting functional difficulty. Well-being refers to how people feel and function at the individual level and with people [10]. It is a measure of quality of life, which means higher well-being is indicative of one’s quality of life. It can be dependent on behaviours such as social and physical activities, which are less frequently performed by older adults in developing countries such as Ghana [11].

Countries such as Ghana where most older adults are unemployed and live in low-socio-economic areas [11] faces a high risk of frailty. Supporting this view is a study that reported an age-standardised frailty prevalence rate of about 38% for Ghana, one of the highest among rates reported for six low- and middle-income countries [12]. As such, advancing research on the effect of frailty on well-being and other health-related variables such as Physical Activity Behaviour (PAB) in Ghana is necessary.

Studies have shown that PAB can be associated with lower frailty [13, 14]. Physical activity is any movement of the body that requires energy expenditure above 1.5 basal metabolic rate [15, 16]. Physical activity examples include running and brisk walking, which can be assessed with either objective or subjective measures. Objective measures include pedometers and accelerometers whereas questionnaires are subjective measures. PAB is a subjective measure of PA that requires individuals to report the activities they performed over the past week. This measure was preferred for this study because it well aligned with our subjective measures of frailty, well-being, and physical function. PAB is a health-seeking behaviour in the sense that it can protect the individual from disease.

Interventions enabling individuals to maintain PAB can be effective at delaying the onset and progression of frailty [14]. There is, nevertheless, a paucity of studies evidencing whether PAB can modify the effect of frailty on health-related variables such as functional difficulty and well-being. Research exists on the relationship between frailty and well-being measures such as quality of life [17, 18], frailty and functional difficulty [13, 14], and frailty and PAB [13, 19]. The evidence from this research suggests that poorer well-being or lower quality of life is associated with higher frailty, PAB can be associated with lower frailty, and higher frailty is associated with higher functional difficulty. Even so, these relationships have been assessed in separate studies or in parts, which conceals potential mediation and moderation effects in them. PAB lowers both frailty and functional difficulty and improves well-being [13, 19], which means it can moderate the indirect association of frailty on well-being through functional difficulty. This potential moderated mediation by functional difficulty in the relationship between frailty, PAB, and well-being implies that the potential negative effect of frailty on well-being can be reversed or weakened by PAB. A confirmation of this moderated mediation would provide evidence for workplace physical activity programmes. This study aimed to test this role, given that no study has investigated it.

Further to the above, individuals with lower income are more vulnerable to frailty and poorer well-being [4, 20–23], so the effect of frailty on well-being may not be the same for older employee groups with unequal income levels. If so, the foregoing moderated mediation role may not be the same for older employee groups with different income levels. Income is one of the individual factors that decline with age, especially in less developed countries where many older adults are poor [11]. As such, policies aimed at improving well-being among older adults with different income levels are needed in less developed countries such as Ghana. An assessment of the above moderated mediation role for older employee groups with unequal income levels can provide evidence for the development or enhancement of these policies.

**Fig. 1:**
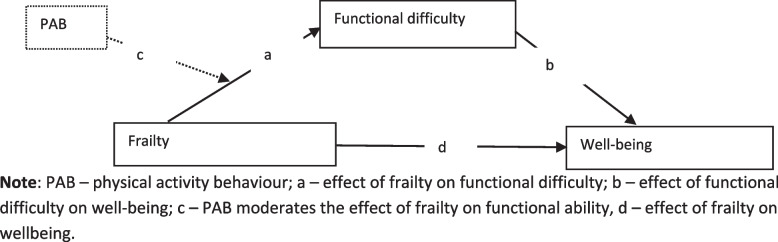
The conceptual framework of path analysis

Given the increasing population ageing, organizations ought to roll out interventions to enable ageing employees to maintain health and productivity by prioritising programmes that can reduce the risk of frailty and its influence on well-being. This viewpoint and the above gaps in the literature informed the current study, which aimed to assess a potential moderated mediation by functional difficulty in the relationship between frailty, PAB, and well-being. PAB serves as the moderator of the effect of frailty on functional difficulty, and functional difficulty serves as a mediator of the link between frailty and employee wellbeing. With this analysis, we aimed to provide evidence by which future researchers and organizations can implement programmes enabling ageing employees to maintain PAB in the interest of employee wellbeing. The following hypotheses were test: (1) frailty has a direct effect on well-being, (2) frailty has an indirect effect on well-being through functional difficulty, and (3) there is a moderated mediation by functional difficulty in the relationship between frailty, PAB, and well-being? These hypotheses were tested with a conceptual model (see Fig. 1).

This study is novel for some reasons. Firstly, it is the first to test a model of the foregoing moderated mediation, which takes previous research beyond an assessment of the direct effects of PAB on frailty and functional difficulty. One of the dominant predictors of frailty and its effects on well-being is income [4, 20, 21], which implies that the moderated mediation may differ between employee groups with different income levels. This study, therefore, assessed the moderated mediation models of two employee groups with different income levels. This analysis provides estimates of the two groups that practitioners and researchers may need and is needed in organizations for understanding implications for employee reward, and health insurance payments. Finally, this study employed a cross-sectional design compliant with the STROBE (e.g., Strengthening the Reporting of Observational Studies in Epidemiology) checklist as well as methods that can guide future research.

## Methods

### Study design

We employed a cross-sectional research design in adherence to the STROBE (Strengthening the Reporting of Observational Studies in Epidemiology) checklist. The cross-sectional design was adopted because our hypotheses involved testing the moderated mediation with data collected at a fixed time. It was suited for our moderated mediation analysis because the data resulting from its use was numeric. Thus, the conceptual moderated mediation model, as illustrated in Fig. 1, was subjected to empirical testing.

### Participants and recruitment

The participants were older employees aged 50 years or older in Accra Ghana. We aimed to include in the study two samples with different income levels. As such, samples were taken from areas of low and high socioeconomic status in Accra, with the low and high socioeconomic areas expected to provide the low- and higher-income samples respectively. The selection criteria applied to both areas were: (1) being aged 50 years or older; (2) being an employee; (3) having a minimum of a basic educational qualification, which evidenced the ability to complete the surveys in English, and (4) being willing and available to complete the surveys. A total of 1765 (low socio-economic areas, *n* = 963, and high socio-economic areas, *n* = 802) employees met these criteria. A structured interview was utilised to find out whether potential participants met the above criteria. Interviews were conducted at community centres and lounges of organizations, and the average time spent in the interviews was 6 min. Incentives were not provided to participants.

The high socioeconomic areas (e.g., East Legon, McCarthy Hill, and Cantonments) predominantly contained senior-level employees including business executives, managers of public and private organizations, and high-profile academics whereas the low-income areas (e.g., Bortianor, Kokompe, and Dodowa) contained junior and middle-level employees of organizations, including petty traders. The high socio-economic areas, unlike the low socio-economic areas, had all amenities as well as tarred and interconnected streets. We utilised the G*Power software and relevant statistics (i.e., effect size = 0.20, power = 0.80, α = 0.05) [24, 25] to calculate the minimum sample necessary for this study. We utilised these statistics and the G*Power software as standards recommended or utilised in the literature [24, 25]. The sample size calculated was 91 for a multiple regression analysis involving a maximum of 10 predictors. We calculated the sample size based on 10 predictors because the maximum number of predictors measured and expected to be incorporated into the moderated moderation model was 10. To maximise the power of our analysis, we gathered data on all eligible participants.

### Measures

Functional difficulty was measured with a 14-item scale adopted in whole with its descriptive anchors (i.e., 1 – not difficult, 2 – somewhat difficult, 3 – most difficult, and 4 – could not perform) from a previous study [26]. This tool measured how difficult it was for individuals to perform physical ADL and IADL tasks over the last week. It produced satisfactory Cronbach’s α on both samples (overall α = 0.87, low-income, α = 0.86, and higher income, α = 0.93). It scores range from 14 to 56, with higher scores indicting higher functional difficulty. This scale was chosen because it was previously validated as a standard measure of functional difficulty and potentially suited our Ghanaian sample.

Frailty was measured with the 15-item Tilburg Frailty Index and its two descriptive anchors (i.e., 0 – no, and 1 – yes) from previous research [5]. This scale was preferred to others because it measures frailty in a clinical context, is relatively short, and is more suited for older adults. It produced satisfactory internal consistency in both samples (overall α = 0.71, low-income, α = 0.72, and higher-income, α = 0.71). Scores of this scale ranges from 0 to 15, with higher scores representing higher frailty. Five items from the World Health Organization’s well-being scale were used to measure well-being [10]. These items, which were used in whole, accompanied five descriptive anchors (i.e., never – 1, sometimes – 2, often – 3, very often – 4, and all the time – 5) and collectively produced satisfactory internal consistency on the two samples (overall α = 0.94, low-income, α = 0.93, and higher income, α = 0.95). Scores of this scale range from 5 to 25, with higher scores indicating higher well-being. We chose this scale because it is a relatively short validated measure and suited our sample of older employees who may not be able complete long surveys.

PAB was measured with a sub-scale from the health-promoting behaviour scale with four anchors (i.e., 1 – never, 2 – sometimes, 3 – often, and 4 – routinely) [15]. This domain of the tool measures how often the individual performed moderate and vigorous physical activities over the last 30 days. It was used for some reasons. First, it is based on short descriptors that could easily be understood and responded to, which meant that older participants with potential memory limitations could complete it without being asked to recall the specific time they spent on PAB. It is brief and is therefore attractive to older participants with potential physical limitations. Finally, we could not meet the cost of using activity trackers, which would have been the ideal measure.

The PAB scale produced satisfactory internal consistency over the two samples (overall α = 0.81, low-income, α = 0.92, and higher income, α = 0.95). Scores on the PAB scale range from 5 to 20, with larger scores indicating higher PAB. All items of the above scales were parcelled or summed up to create data on the variables in harmony with previous research [27]. Each scale produced a Cronbach’s alpha coefficient ≥ 0.7, which means that items of each scale had sufficient homogeneity or internal consistency. As such, the items could be parcelled or summed up to form a unit. Appendix A shows all scales and items used.

Seven other variables were measured as potential covariates following previous research [25, 27]. Sex (i.e., men – 1, and women – 2), chronic disease status (i.e., 1 – none, and one or more – 2), marital status (i.e., 1 – not married, and 2 – married), and self-reported health (i.e., poor – 1, and good – 2) were measured as categorical variables. Income, age, and education were measured as discrete variables. Income was the individual’s reported gross monthly income in Ghana cedis (₵) whereas education was the individual’s years of schooling. Age was the individual’s chronological age.

### Instrumentation and common methods bias assessment

A self-reported questionnaire comprising two main sections was used to collect data. The first section presented measures of frailty, functional difficulty, PAB, and well-being whereas the second section captured the covariates and personal factors. To avoid or minimise Common Methods Bias (CMB), scales on the questionnaire were randomly arranged and separated with preambles containing survey-completing instructions. Based on Harman’s one-factor method [28, 29], we assessed the factor structures of all scales with exploratory factor analysis through the maximum likelihood method. Frailty and functional difficulty produced 4 and 5 factors in the low-income and higher-income samples respectively, with the first factors extracted associated with a variance < 40% as recommended [28]. The analysis yielded 1 or 2 factors for PAB and well-being in the two samples, and the variance of the first factor was not less than 40%. This factor solution was confirmed with confirmatory factor analysis. Since PAB and well-being were measured with brief scales of only 5 items each, this result was acceptable [28, 30].

### Ethical considerations and data collection

This study received ethical review and clearance from the ethics review committee of the Africa Centre of Epidemiology (number: 001–2023-ACE) after the board reviewed the study protocol. Every participant provided written informed consent after being informed about the purpose and nature of the study. Some of the researchers, supported by trained research assistants, coordinated data collection. Self-reported questionnaires were administered from February 1 to March 3 2023 in organizations, malls, and community centres where the participants were recruited. Because all respondents had formal education, they could complete the questionnaire without the assistance of the researchers and research assistants. Some participants returned completed questionnaires instantly whereas others returned them in two weeks. All questionnaires were returned in about four weeks. A potential source of bias was the possibility of participants understanding and interpreting the questions differently. A total of 1565 (low socio-economic areas, *n* = 956, and high socio-economic area, *n* = 614) were returned, but 1542 were analysed as 200 (low socio-economic areas, *n* = 188, and high socio-economic area, *n* = 12) were discarded for being completed partially.

We used an independent samples t-test to compare the incomes of the two samples. This analysis showed that the higher socio-economic area (Mean = 2540.79; standard deviation = 108.26) had a larger average income compared to the low socio-economic area (Mean = 1111.39; standard deviation = 25.36) at a statistical significance of *p* < 0.001 (t = -12.57, two-tailed). Thus, the low-income sample [i.e., a sample from a low socioeconomic area) had lower income compared to the higher-income sample (i.e., the sample from high socio-economic area). This result enabled us to analyse the data based on our assumption of unequal incomes between the two samples.

### Statistical data analysis

Data were analysed with SPSS 29 (IBM Inc., New York, USA) and Amos; SPSS was used to summarise the data and perform a sensitivity analysis for confounding variables whereas Amos was used to test the moderated-mediation model. The analysis was in two phases, and in the first phase of the analysis, categorical and discrete variables were summarised with frequencies and averages. Gender, marital status, and self-reported health included missing data, but none of these variables had more than 10% missing data and the 'missing at random test' test allowed us to employ the multiple inputation method to replace missing data.

We followed previous research [25, 27] to screen the measured covariates for the “ultimate confounders”, which are measured covariates more likely to confound the primary effects [29, 31]. We performed this analysis with hierarchical linear regression analysis following a previous study [31] to ensure that no irrelevant covariate was incorporated into the model tested. None of the variables qualified as an ultimate covariate after performing this analysis on the two samples. Appendix B1 shows the steps taken in this analysis.

**Fig. 2:**
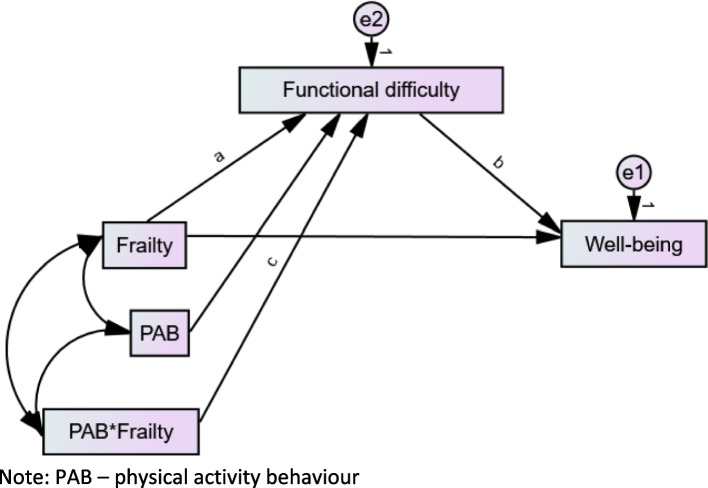
Statistical model of the moderated mediation by functional difficulty

In the second phase of the analysis, the moderated mediation model was fitted with Amos on both samples based on Haye’s Process [32]. Figure 2 shows the statistical moderated mediation model tested. In fitting the model, the moderating variable (i.e., PAB) was mean-centred and the interaction of the centred variable with frailty (i.e., PAB*frailty) was generated using the compute function in SPSS. Relevant path coefficients (a, b, and c in Fig. 2) as well as indexes of moderated mediation were computed with the “user-defined estimands” function in Amos. The specific indexes estimated were the Simple Slopes (SS), Conditional Indirect Effects (CIE), and the Index of Moderated Mediation (i.e., InModMed).

**Fig. 3:**
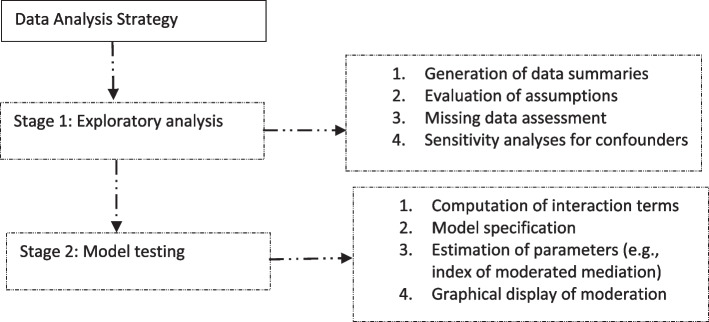
A framework of the statistical analysis method

The index of moderated mediation was a function of the SS and CIE at three levels (i.e., low, middle, and high) of the moderating variable. Appendix B2 shows the formulae used to compute these indexes. Calculations were based on a maximum likelihood estimation and 2000 bias-corrected sampling iterations (through bootstrapping within a 95% confidence interval). The statistical significance of the estimates was detected at a minimum of *p* < 0.05. The moderation effect was visualized with figures showing the relationship between frailty and functional difficulty at low, moderate, and high PAB. Multivariate normal distribution of the data, an assumption for structural equation modelling and Haye’s Process, was not met but this was corrected with the above bias-corrected sampling iterations [33]. Figure 3 is a flowchart of the statistical analysis method.

## Results

In Table 1, 52% (*n* = 492) and 51% (*n* = 304) of the participants were men in the low- and higher-income samples respectively. The average ages of the participants in the low- and higher-income samples were respectively 61 (Mean = 61.31; SD = 40.39) and 60 (Mean = 60.41; SD = 8.86) years. Thus, the average ages of the samples are about the official retirement age of 60 years in Ghana. The averages of frailty in the low- and higher-income samples were about 6 (Mean = 5.56; SD = 3.13) and 7 (Mean = 6.73; SD = 2.70) respectively. The maximum frailty score that one could report on the scale used in this study was 15, which means the above averages represent 37% and 45% of frailty in the low- and higher-income samples respectively. Thus, frailty in both samples was low.

**Table 1:** Summary statistics on variables included in the study

Variable	Group	n/mean	%/SD	n/mean	%/SD
		Low income (*n* = 942)		Higher income (*n* = 600)	
Categorical variables					
Gender	Men	492	52.23	304	50.67
	Women	426	45.22	296	49.33
	Missing	24	2.55	---	---
	Total	942	100.00	600	100.00
Chronic disease status	None	135	14.33	320	53.33
	≥ 1	807	85.67	280	46.67
	Total	942	100.00	600	100.00
Marital status	not married	252	26.75	112	18.67
	married	609	64.65	476	79.33
	Missing	81	8.60	12	2.00
	Total	942	100.00	600	100.00
Self-reported health	poor	303	32.17	248	41.33
	good	582	61.78	348	58.00
	Missing	57	6.05	4	0.67
	Total	942	100.00	600	100.00
Discrete/continuous variables					
Frailty	---	5.56	3.13	6.73	2.70
Physical activity behaviour	---	10.51	2.85	10.66	3.13
Functional difficulty	---	22.28	8.81	22.30	7.99
Well-being	---	13.53	4.09	14.64	4.75
Income (₵)	---	1111.39	25.36	2540.79	108.26
Age (yrs)	---	61.31	40.39	60.41	8.86
Education (yrs)	---	21.32	4.21	21.12	3.02

*n* frequency, *SD* standard deviation; frequency and % apply to categorical variables whereas mean and SD apply to discrete or continuous variables

In Table 2, PAB has a positive effect on functional difficulty in the low-income (β = 0.267; critical ratio = 3.607; *p* < 0.001) and higher-income sample (β = 0.432; critical ratio = 3.946; *p* < 0.001). Thus, higher PAB was associated with higher functional difficulty in both samples. Frailty has a positive effect on functional difficulty but has a negative effect on well-being in both samples, which suggests that employees with higher frailty reported higher functional difficulty but lower well-being.

**Table 2:** Association of frailty and functional difficulty with well-being in the two samples

Dependent variable	Path	Direct effects							Indirect effects	
		Predictor	B	β	SE (of B)	Critical ratio	*p*	Label	β	*p*
Low-income sample (*n* = 942)										
Functional difficulty	< ---	PAB	0.826	0.267	0.229	3.607	***			
Functional difficulty	< ---	Frailty	0.759	0.269	0.090	8.428	***	a		
Well-being	< ---	Frailty	-0.179	-0.137	0.043	-4.125	***		-0.021	0.009
Well-being	< ---	Functional difficulty	0.052	0.112	0.015	3.354	***	b		
Functional difficulty	< ---	PAB*Frailty	-0.084	-0.192	0.032	-2.600	0.009	c		
Higher income sample (*n* = 600)										
Functional difficulty	< ---	PAB	1.103	0.432	0.280	3.946	***			
Functional difficulty	< ---	Frailty	0.748	0.253	0.111	6.761	***	a		
Well-being	< ---	Frailty	-0.753	-0.428	0.059	-12.657	***		-0.085	***
Well-being	< ---	Functional difficulty	-0.199	-0.335	0.020	-9.905	***	b		
Functional difficulty	< ---	PAB*Frailty	-0.292	-0.724	0.044	-6.625	***	c		

B – unstandardized regression weight (effect); β – standardised regression weight (effect); *PAB* physical activity behaviour, *SE* standard error; the variances explained in the dependent variable in the low and higher-income samples are 56.3% and 58.1% respectively

^***^p < 0.001

**Fig. 4:**
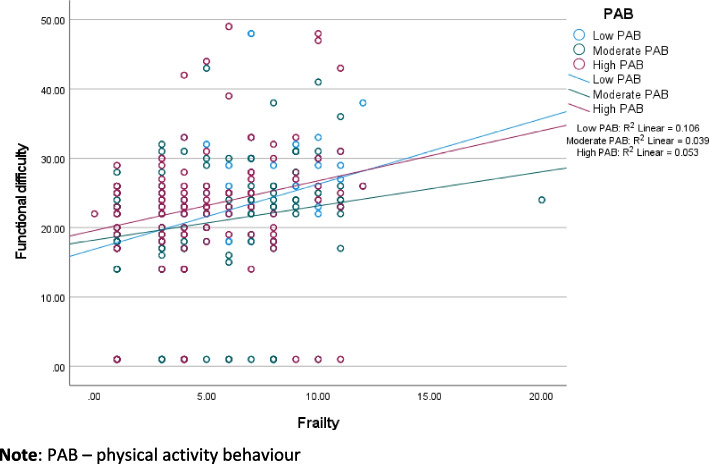
The effect of frailty on functional difficulty at diferent levels of physical activity beahviour (low-income sample, *n* = 942)

**Fig. 5:**
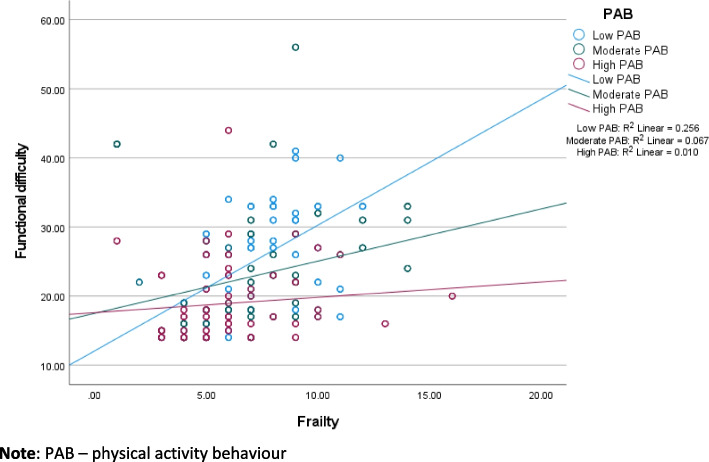
Effect of frailty on functional difficulty at diferent levels of physical activity behaviour (higher income sample, *n* = 600)

In Table 2, frailty has an indirect negative effect on well-being through functional difficulty in the low-income (β = -0.021; *p* < 0.05) and higher-income (β = -0.085; *p* < 0.001) samples. The interaction between frailty and PAB (i.e., PAB*frailty) has a positive effect on functional difficulty in both samples. The regression weights between frailty and functional difficulty in the low- and higher-income samples are respectively β = 0.269 and β = 0.253. The regression weights of PAB*frailty in both samples are negative (low-income β = -0.192, and higher-income β = -0.724), which implies that lower functional difficulty is associated with frailty at higher PAB. Figures 4 and 5 visualize the interaction of PAB and frailty on functional difficulty. In the low-income sample, frailty is less strongly associated with functional difficulty at moderate (compared to low) PAB (see Fig. 4). In the higher income sample, frailty is less strongly associated with functional difficulty at higher PAB.

In Table 3, the moderated mediation indexes are statistically significant in both the low-income (β = -0.004; *p* < 0.05) and higher-income (β = 0.058; *p* < 0.001) samples. Thus, the negative indirect effects of frailty on well-being through functional difficulty are lower at higher levels of PAB. In the higher-income sample, the indirect effect of frailty on well-being through functional difficulty is positive at higher PAB. This result implies that the moderated mediation by functional difficulty may better support well-being in the higher-income sample, compared to the low-income sample.

**Table 3:** Estimates of moderated mediation by functional difficulty in the two samples

Parameter	Low-income sample (*n* = 942)				Higher income sample (*n* = 600)			
	B	95% CI		*p*	B	95% CI		*p*
		Lower	Upper			Lower	Upper	
Estimates of simple slopes at three levels of PAB								
LowSS	0.999	0.777	1.23	***	1.662	1.283	2.072	***
MidSS	0.759	0.572	0.951	***	0.748	0.506	1.015	***
HighSS	0.518	0.213	0.803	***	-0.166	-0.437	0.127	0.238
Estimates of conditional indirect effects at three levels of PAB								
LowCIE	0.052	0.015	0.093	0.004	-0.331	-0.436	-0.241	***
MidCIE	0.039	0.011	0.073	0.004	-0.149	-0.214	-0.096	***
HighCIE	0.027	0.006	0.057	0.005	0.033	-0.025	0.085	0.238
Estimate of moderated mediation								
InModMed	-0.004	-0.009	-0.001	0.009	0.058	0.043	0.077	***

B – unstandardised regression weight; *SS* simple slope, *CIE* conditional indirect effect, *CI* confidence interval (based on 2000 bias corrected sampling iterations), *InModMed* index of moderated mediation, *PAB* physical activity behaviour; please refer to Appendix B2 for formulae used to calculate the simple slopes and conditional indirect effects

^***^p < 0.001

## Discussion

This study investigated whether there is a moderated mediation by functional difficulty in the relationship between frailty, PAB, and well-being. Models of the moderated mediation were compared between low- and higher-income samples.

The study confirmed a direct negative effect of frailty on well-being in both samples, though this effect is stronger in the higher-income sample. This result suggests that older adults with higher frailty reported lower well-being in both samples, which is consistent with several studies conducted around the world [17, 18, 34]. A systematic review in the United Kingdom [17] found a negative association between frailty and quality of life, which is an indicator of well-being. Though quality of life and well-being are unique measures, the result reported in the above systematic review significantly aligns with our evidence for some reasons. Firstly, quality of life and well-being are recognised in the literature as related indicators of health. Secondly, measures of quality of life, such as the 36-item short-form quality of life tool, include measures of perceived well-being [17].

In France, another systematic review found a negative relationship between frailty and well-being [34] and thus supports our result. This systematic review, compared to the above systematic review conducted in the United Kingdom, better aligns with our result, since it focused on our dependent variable (i.e., well-being). In Sri Lanka, a negative association between frailty and quality of life was found. The current study builds on the above pieces of evidence by confirming the negative frailty-well-being link between two samples with different income levels. Previous studies [4, 21] have found that frailty is higher in older adults with lower income, but the effect of frailty on well-being was not compared between low- and higher-income samples.

This study further confirmed an indirect negative effect of frailty through functional difficulty on well-being, which suggests that frailty had a positive effect on functional difficulty, which in turn had a negative effect on wellbeing. Thus, higher frailty was associated with higher functional difficulty as reported in previous research [35, 36], and functional difficulty was associated with poorer self-reported wellbeing. In harmony with our evidence regarding the link between functional difficulty and well-being, researchers [17, 34, 36] have acknowledged that older adults with functional limitation or difficulty would report poor well-being or health, possibly owing to physiological limitations and pains accompanied by functional limitation. Since frailty has a direct effect on well-being, the above mediation is partial [32, 37], signifying that frailty, apart from its direct influence on poorer well-being, can be associated with poorer well-being through functional difficulty.

There was a moderated mediation by functional difficulty in the relationship between frailty, PAB, and well-being. This result has two main parts, the first being the moderating role of PAB in the relationship between frailty and functional difficulty. This moderation implies that frailty was less negatively associated with functional difficulty at higher PAB. Suffice it to say that the maintenance of PAB may be associated with a lower chance of frailty predicting functional difficulty. Several studies [13, 14, 19, 38] have shown that people are less likely to be frail if they perform PAB, especially over time [14]. The current study extends this evidence by showing that frailty is less likely to be associated with higher functional difficulty at higher PAB. In both samples, frailty rather buffers functional difficulty at higher PAB, though this relationship is stronger in the higher income sample. Thus, this study reinforces the role of PAB in buffering the risk of functional difficulty in both samples.

The moderated mediation suggests that the foregoing mediation is moderated by PAB. A moderated mediation involves four variables that form a nexus. The basic element of this nexus is the direct effect of frailty on well-being. In this study, functional difficulty as the mediating variable transmits the effect of frailty to well-being, which signifies the indirect effect of frailty on well-being in both samples. The final element of this relationship is frailty having different sizes of effect on functional difficulty at different levels of PAB. Similarly, the size of the indirect effect changes as PAB increases. The moderated mediation in practice implies that the indirect negative effect of frailty through functional difficulty on well-being is smaller at higher levels of PAB. Alternatively, frailty is less likely to predict poorer well-being through functional difficulty at higher PAB, which emphasises the role of PAB in preserving well-being by lowering the risk of frailty and functional difficulty. Noteworthy is the difference between the low- and higher-income samples concerning the direct effect of frailty on well-being, mediation effect, and moderated mediation effect. These effect sizes are not the same for the two samples, which suggests that the extent to which frailty predicts well-being through functional difficulty and PAB (in the context of a moderated mediation) can vary between older employee groups with different levels of income. This result is an extension of previous evidence regarding the relationship between income and frailty [4, 21].

The role of PAB in the moderated mediated model helps to build upon the Activity Theory of Ageing, which argues that individuals can maintain PAB, health, and functioning as they age [25, 39]. This ability to maintain PAB comes from adaptive behaviour or the capacity to adapt previous life experiences. The current evidence suggests that the maintenance of PAB does not only directly buffer the risk of frailty and functional limitation as suggested by previous studies [35, 36] but also indirectly predicts better well-being in the context of its moderated mediation. Our results, thus, imply a need for employers and organizations to support their employees to maintain PAB as a health behaviour. Any policy that guides organizations to invest in workplace PAB promotion programmes may contribute to profitability by improving well-being and reducing the cost of staff absences from work due to ill health.

The negative effect of frailty on functional difficulty provides implications for clinical planning. This effect means in practice that frail older adults are more likely to experience functional limitations characterised by a low ability to perform physical tasks. Thus, in situations where clinical frailty is high, the burden of functional limitation and its underlying healthcare needs may be high, an idea supported by researchers [2, 22]. As such, interventions enabling older adults to avoid or delay the onset of frailty may lower the risk of functional limitations among older adults. These interventions may, therefore, reduce hospitalisations related to frailty.

Our results regarding the moderation role of PAB add to this understanding with its implication that the burden of healthcare due to frailty-related functional limitation may be higher among those with higher income. Considering the interaction between PAB and frailty on functional difficulty, it can be said that PAB is more likely to buffer the negative influence of frailty on functional difficulty in the higher-income sample. Similarly, frailty is less likely to be associated with poorer well-being at higher PAB in the higher-income sample. Deductively, employers may reduce frailty-related functional limitation and sick leaves due to poor well-being by rolling out policies to support employee PAB. Such policies may be more beneficial to employees with higher income.

Research to date [1, 40] suggests that the burden of disease and its associated healthcare may be higher in frail populations. The current study builds on this understanding by providing evidence on how frailty may predict functional difficulty, a major factor influencing the burden of healthcare [9, 41], at different PAB levels and in groups with different income levels. Specifically, the burden of healthcare among people with frailty can be assessed based on differences in PAB and income among employees. Health service providers may face a higher burden of frailty-driven care from groups with lower income, more so in groups with little or no PAB. This means organizations may lose more money to health insurance payments linked to frailty-related healthcare among those with low pay and PAB. This point explains why minimising inequality in pay and enabling employees to maintain PAB at the organizational level can save financial resources and optimise profitability.

Worth noting are the differences in effect sizes between the low- and higher-income samples. The higher-income sample produced larger effect sizes for all paths, except for the path between functional difficulty and frailty. For example, the effect of the interaction term (i.e., PAB*Frailty) on functional difficulty is 277% stronger in the high-income sample. These observed differences have some practical implications. A larger interaction effect in the higher-income sample implies that PAB would more strongly reduce or reverse the positive effect of frailty on functional difficulty among older adults with higher income. Alternatively, frailty is less likely to result in functional difficulty at higher PAB among older adults with higher income, compared with those with low income. Even so, these differences need to be investigated in other populations to assess their consistency across contexts.

### Strengths and limitations

We acknowledge that this study has some limitations. This study could not establish causation between the variables with its cross-sectional design, which means the effect sizes reported should ideally be viewed as associations. In view of this shortcoming of the study, we call for future studies that can establish causation. The generalisability of our findings may be limited by two factors. The first is our adoption of a non-probability sampling technique which could not enable us to reach a representative sample. Moreover, our samples were limited to Accra, Ghana, so we call for future studies utilising probabilistic and representative samples. Subjective (self-reported) measures were used, so the results of the study were susceptible to response bias. Since we measured PAB with a standard questionnaire, future researchers are encouraged to use physical activity trackers or any other measure that overrides recall bias.

Our study touches upon the relationship between income levels and frailty. However, the complex interplay of socioeconomic factors in health outcomes is a multifaceted issue. Some researchers [23, 42] contend that it is not merely income but also factors such as education, access to healthcare, and occupational conditions that significantly contribute to health disparities. Our study focuses on income, but it is crucial to consider the broader socioeconomic context and how its multiple factors may interact in influencing well-being and functional difficulty. Engaging in a dialogue on the multifactorial nature of health disparities and the potential role of policy interventions could provide a broader perspective on addressing the health needs of older adults across different income groups.

Despite the above limitations, this study is significant for several reasons. Apart from being the first to test the moderated mediated model, it focuses on older employees, a population not previously studied. With the increasing rate of population ageing around the world and a need for organizations to roll out workplace ageing policies, a focus on this segment of the population was significant. This study’s design is stronger than an average cross-sectional design in several ways. It is compliant with the STROBE [43] and includes a sensitivity analysis aimed at curbing potential confounding. Appendix C shows items of the STROBE checklist met. Our utilisation and comparison of two income-variant samples enhanced the robustness of our statistical analysis. The moderated mediation was based on 2000 bias-corrected sampling iterations (at a 95% confidence interval), which could minimise sampling bias.

## Conclusion

Older employees with higher frailty reported lower well-being in the low- and higher-income samples, though frailty more strongly predicted lower well-being in the higher-income sample. Frailty had a negative indirect effect on well-being through functional difficulty in both samples, but this effect was larger in the higher-income sample. The mediation of functional difficulty in the association between frailty and well-being was partial in both samples. PAB moderated the positive association of frailty with functional difficulty, which suggests that employees with higher frailty reported lower functional difficulty at higher PAB. This moderation role was stronger in the higher-income sample, which means that PAB more strongly buffered the effect of frailty on functional difficulty in the higher-income sample. The negative indirect effect of frailty on well-being in both samples was lower at higher PAB. We conclude that PAB may buffer the positive effect of frailty on functional difficulty and that the indirect negative effect of frailty on well-being may be weaker at higher PAB. Organizations are encouraged to roll out workplace interventions aimed at encouraging PAB.

## Supplementary Information

Supplementary Material 1.

Supplementary Material 2.

Supplementary Material 3.

Supplementary Material 4.

Supplementary Material 5.

## Data Availability

Data used for this study are available as an online supplementary material (i.e., data_higher_income and data_lower_income).
