# Supplementary material for: Association of frailty with physical activity behaviour and well-being in older employees: moderated mediation by functional difficulty
Source: BMC Public Health. 2025 Jan 31;25:400. doi: 10.1186/s12889-025-21596-9 (PMC11786485; doi:10.1186/s12889-025-21596-9)
Supplement: Supplementary file 1 — Supplementary Material 1. [file 12889_2025_21596_MOESM1_ESM.doc]

Appendix A. Scales used to measure frailty, functional difficulty, well-being, and physical activity behaviour

**Appendix A1. Items for measuring functional difficulty**

| No. | Task/item | Response | | | |
| --- | --- | --- | --- | --- | --- |
|  |  | 1 | 2 | 3 | 4 |
| 1 | Lifting weight (up to 1–2 kg) |  |  |  |  |
| 2 | Bending |  |  |  |  |
| 3 | Squatting |  |  |  |  |
| 4 | Walking (up to 1–2 km) |  |  |  |  |
| 5 | Climbing |  |  |  |  |
| 6 | Arising from bed/chair |  |  |  |  |
| 7 | Household work |  |  |  |  |
| 8 | Outside work |  |  |  |  |
| 9 | Use of public transport |  |  |  |  |
| 10 | Social gathering |  |  |  |  |
| 11 | Self-cleaning (Bathing) |  |  |  |  |
| 12 | Toilet use |  |  |  |  |
| 13 | Dressing |  |  |  |  |
| 14 | Eating food |  |  |  |  |

Note: descriptive anchors 1 – no difficult, 2 – somewhat difficult, 3 – most difficult, and 4 – unable to perform.

**Appendix A2. Items for measuring frailty**

| No. | Question | Response | |
| --- | --- | --- | --- |
|  |  | No (0) | Yes (1) |
| 1 | Do you feel physically healthy? |  |  |
| 2 | Have you lost a lot of weight recently without wishing to do so? |  |  |
| 3 | Do you experience problems in your daily life due to difficulty in walking? |  |  |
| 4 | Do you experience problems in your daily life due to difficulty maintaining your balance? |  |  |
| 5 | Do you experience problems in your daily life due to poor hearing? |  |  |
| 6 | Do you experience problems in your daily life due to poor vision? |  |  |
| 7 | Do you experience problems in your daily life due to a lack of strength in your hands? |  |  |
| 8 | Do you experience problems in your daily life due to physical tiredness? |  |  |
| 9 | Do you have problems with your memory? |  |  |
| 10 | Have you felt down during the last month? |  |  |
| 11 | Have you felt nervous or anxious during the last month? |  |  |
| 12 | Are you able to cope with problems well? |  |  |
| 13 | Do you live alone? |  |  |
| 14 | Do you sometimes miss having people around you? |  |  |
| 15 | Do you receive enough support from other people? |  |  |

**Appendix A3. Items for measuring well-being**

| # | Item | 1 | 2 | 3 | 4 | 5 |
| --- | --- | --- | --- | --- | --- | --- |
| 1 | I have felt cheerful and in good spirits |  |  |  |  |  |
| 2 | I have felt calm and relaxed |  |  |  |  |  |
| 3 | I have felt active and vigorous |  |  |  |  |  |
| 4 | I woke up feeling fresh and rested |  |  |  |  |  |
| 5 | My daily life has been filled with things that interest me |  |  |  |  |  |

Note: descriptive anchors are never – 1; sometimes – 2; often – 3, very often – 4, and all the time – 5.

**Appendix A4. Items for measuring physical activity behaviour**

| No | Statement | 1 | 2 | 3 | 4 |
| --- | --- | --- | --- | --- | --- |
| 1 | Exercised vigorously for 20 or more minutes at least three times a week (such as brisk walking, bicycling, aerobic dancing, and using a stair climber) |  |  |  |  |
| 2 | Took part in light to moderate physical activity behaviour (such as walking 30-40 minutes 5 or more times a week. |  |  |  |  |
| 3 | Took part in leisure-time (recreational) physical activities (such as dancing, swimming, and bicycling) |  |  |  |  |
| 4 | Did stretching exercises at least 3 times per week. |  |  |  |  |
| 5 | Got exercise during usual daily activities (such as walking during lunch, using stairs instead of elevators, and parking car away from destination and walking). |  |  |  |  |

Note: Descriptive anchors are 1 – never, 2 – sometimes, 3 – often, and 4 – routinely
