# Supplementary material for: Association of frailty with physical activity behaviour and well-being in older employees: moderated mediation by functional difficulty
Source: BMC Public Health. 2025 Jan 31;25:400. doi: 10.1186/s12889-025-21596-9 (PMC11786485; doi:10.1186/s12889-025-21596-9)
Supplement: Supplementary file 2 — Supplementary Material 2. [file 12889_2025_21596_MOESM2_ESM.doc]

Appendix B

**Appendix B1. Procedures followed to perform the sensitivity analysis for confounders**

| Stage | # | Action (performed on each of the two samples) |
| --- | --- | --- |
| 1 | 1 | Fit a simple linear regression model to assess the relationship between frailty and wellbeing |
|  | 2 | Note the standardised regression weight from step 1 |
|  | 3 | Fit a multiple linear regression model in which all measured confounding variables are treated as predictors of frailty, the main predictor |
|  | 4 | Identify from step 3 potential confounders that have a p-value ≥0.25 |
|  | 5 | Predictors from step 4 that produced a p≥0.25 should be removed from the analysis and the others kept for the next stage of the analysis |
| 2 | 6 | Adjust for each of the remaining confounding variables in the model fitted at step 1 |
|  | 7 | Compute the per cent (%) change between the standardised regression weight at step 1 and the new weight resulting from step 6 |
|  | 8 | All potential confounders that produce a change of 10% or more should be incorporated into the final analysis as the ultimate confounders |

**Appendix B2. Equations for estimating the index of moderated mediation at three stages**

**Stage 1: estimation of simple slopes based on the mean-centred moderated**

Key statistics from the data:

1. mean of centred moderator (i.e., physical activity behaviour) for low-income sample = 0
2. standard deviation (SD) of the moderator for the low-income sample = 2.83683
3. mean of centred moderator for higher-income sample = 0
4. standard deviation of the moderator for the higher-income sample = 3.12848

**General equations**

lowSS=a+c*(mean – 1SD)

medSS=a+b*(mean)

highSS=a+c*(mean + 1SD)

**Equations based on data from the low-income sample**

lowSS=a+c*(-2.83683)

medSS=a

highSS=a+c*(2.83683)

**Equations based on data from the higher-income sample**

lowSS=a+c*(-3.12848)

medSS=a

highSS=a+c*(3.12848)

**Stage 2: estimation of the conditional indirect effects based on estimated simple slopes**

lowCIE=lowSS*b

medCIE=medSS*b

highCIE=highSS*b

**Stage 3: estimation of the index of moderated mediation**

InModMed=c*b

Note: please see the statistical model tested to make sense of these equations. * represents multiplication
